# Combined Role of Spirulina and Exercise-Based Interventions in Individuals with Overweight and Obesity: A Systematic Review and Meta-Analysis

**DOI:** 10.3390/jcm15062137

**Published:** 2026-03-11

**Authors:** Yavuz Yasul, Taner Akbulut, Vedat Çınar, Muhammet Enes Yasul, Gian Mario Migliaccio, Do-Youn Lee

**Affiliations:** 1 Bafra Vocational School, Ondokuz Mayıs University, 55400 Samsun, Türkiye; yavuz.yasul@omu.edu.tr; 2 Department of Coaching Education, Faculty Sport Science, Fırat University, 23119 Elazig, Türkiye; 3 Department of Physical Education and Sport, Faculty Sport Science, Fırat University, 23119 Elazig, Türkiye; cinarvedat@hotmail.com; 4 Department of Physical Education and Sport, Institute of Health Science, Fırat University, 23119 Elazig, Türkiye; muhammetenes.yasul@saglik.gov.tr; 5 Department of Human Sciences and Promotion of the Quality of Life, San Raffaele Rome Open University, 00166 Rome, Italy; gianmario.migliaccio@uniroma5.it; 6 College of General Education, Kookmin University, Seoul 02707, Republic of Korea

**Keywords:** spirulina, exercise, physical activity, overweight, obesity, VO2max, lipid profile

## Abstract

**Background**: Spirulina supplementation combined with structured exercise may improve obesity-related metabolic dysfunctions. This research examined whether this combination enhances body composition, glucose levels, lipid profile, and cardiorespiratory fitness in overweight and obese adults. **Methods**: Following PRISMA 2020 guidelines, a systematic search of Scopus, PubMed, and Web of Science identified randomized controlled trials (RCTs) evaluating spirulina (1–6 g/day) combined with structured exercise in individuals with overweight and obesity (BMI ≥ 25). The search retrieved 91 records, of which 10 studies met the inclusion criteria and were included in the systematic review. Nine studies provided sufficient post-intervention data and were included in the quantitative meta-analysis using a random-effects model, with heterogeneity assessed using τ^2^, Q, and I^2^ statistics. Publication bias was evaluated using rank correlation, regression-based tests, trim-and-fill, and fail-safe N analyses. **Results**: Combined spirulina supplementation and structured exercise (6–12 weeks) was associated with reductions in BMI (−1.34 kg/m^2^), body fat percentage (−3.03%), fasting glucose (−14.47 mg/dL), LDL-C (−12.68 mg/dL), and triglycerides (−9.81 mg/dL), along with increases in VO2max (3.25 mL/kg/min) and HDL-C (4.21 mg/dL). Effect estimates were generally larger in combined exercise–spirulina subgroups, particularly in HIITsupp and R-AEsupp conditions, whereas supplementation-only comparisons demonstrated smaller and less consistent changes. Inflammatory markers and adipokines (CRP, TNF-α, MCP-1, IL-6, IL-8) showed favorable directional changes in individual trials. **Conclusions**: Spirulina combined with structured exercise was associated with changes in anthropometric, glycemic, cardiorespiratory, and lipid parameters in individuals with overweight or obesity.

## 1. Introduction

Obesity constitutes a major global health concern, closely associated with a range of metabolic dysfunctions such as insulin resistance, dyslipidemia, and chronic systemic inflammation. These dysfunctions significantly elevate the risk of developing type 2 diabetes, cardiovascular disease, and other metabolic syndromes, underscoring the need for comprehensive and integrative interventions [1,2]. According to the World Health Organization (WHO), individuals with a body mass index (BMI) ≥ 25 kg/m^2^, encompassing both overweight (25.0–29.9 kg/m^2^) and obese (≥30.0 kg/m^2^) categories, are at increased cardiometabolic risk and were therefore included in this systematic review. Structured exercise, defined as a planned, supervised, and progressively overloaded physical training regimen with specified frequency, intensity, and duration, was a central intervention across the included studies. This term covered diverse exercise modalities, including both moderate-intensity continuous training and high-intensity interval training. Although the beneficial effects of exercise alone are well established, a growing body of evidence suggests that combining exercise with nutritional interventions, such as spirulina supplementation, may yield enhanced improvements in metabolic health outcomes [3,4].

Among various functional nutrients, spirulina, a cyanobacterium rich in bioactive compounds, has been extensively studied for its antioxidant, anti-inflammatory, lipid-lowering, and insulin-sensitizing properties [5]. Spirulina supplementation has been reported to modulate lipid profiles, improve insulin sensitivity, and reduce systemic inflammation in individuals with overweight or obesity, as reported in previous human studies [6,7]. However, despite the individual benefits of exercise training and spirulina supplementation, research examining their combined effects on metabolic health remains limited and fragmented [8,9].

According to the systematic review by Weston et al. [10], structured exercise interventions, particularly high-intensity interval training, are effective in improving cardiometabolic health in individuals with overweight and obesity. In line with these findings, two randomized controlled trials have investigated the combined effects of spirulina supplementation (1–6 g/day) and structured exercise training on BMI, body fat percentage, lipid metabolism, inflammatory markers, and cardiovascular fitness [11,12]. These trials demonstrate methodological variability, including differences in spirulina dosage, intervention duration (6–12 weeks), exercise modality (aerobic training, resistance–aerobic combinations, HIIT, or circuit resistance training), and outcome selection. In some instances, multiple publications originate from the same participant cohort but examine different exercise conditions. Such variation limits direct comparability and contributes to uncertainty regarding the magnitude of combined intervention effects. Therefore, a systematic evaluation of existing evidence is necessary to determine whether spirulina is associated with exercise-induced metabolic and physiological adaptations in sedentary and metabolically at-risk populations [13,14].

The present study aimed to systematically evaluate whether spirulina supplementation combined with structured exercise is associated with differences in anthropometric, glycemic, lipid, inflammatory, and cardiorespiratory outcomes compared with exercise alone. Specifically, we evaluated whether the combined intervention was associated with changes in BMI, body fat, and insulin resistance, including homeostatic model assessment (HOMA-IR), fasting insulin, and glucose and lipid markers, including high-density lipoprotein (HDL), low-density lipoprotein (LCL) and triglycerides, along with anti-inflammatory effects, including C-reactive protein (CRP), tumor necrosis factor-alpha (TNF-α), monocyte chemoattractant protein-1 (MCP-1), interleukin-6 (IL-6), interleukin-8 (IL-8) and improved cardiovascular fitness (VO2max). Although the individual effects of spirulina or exercise have been previously reviewed, no systematic review or meta-analysis has specifically synthesized evidence regarding their combined impact in adults classified as overweight or obese. This study addresses that gap by critically evaluating randomized controlled trials (RCTs) that investigate this dual intervention and by clarifying inconsistencies reported in the existing literature.

## 2. Materials and Methods

### 2.1. Study Design

This systematic review and meta-analysis were conducted in accordance with the PRISMA 2020 guidelines (Supplementary Materials) [15]. The study protocol was registered in the PROSPERO database under the ID number 1034087. Systematic literature searches were performed in PubMed, Scopus, and Web of Science databases from 1 October 2024 to 30 December 2024. The PubMed search strategy combined Medical Subject Headings (MeSH) with free-text terms using Boolean operators (AND/OR), including the keywords *spirulina*, *exercise*, *physical activity*, *overweight*, *obesity*, and *obese*. Searches were restricted to peer-reviewed, full-text articles published between 2014 and 2024 to ensure the inclusion of methodologically sound and clinically relevant evidence.

### 2.2. Study Selection Process

**Figure 1:**
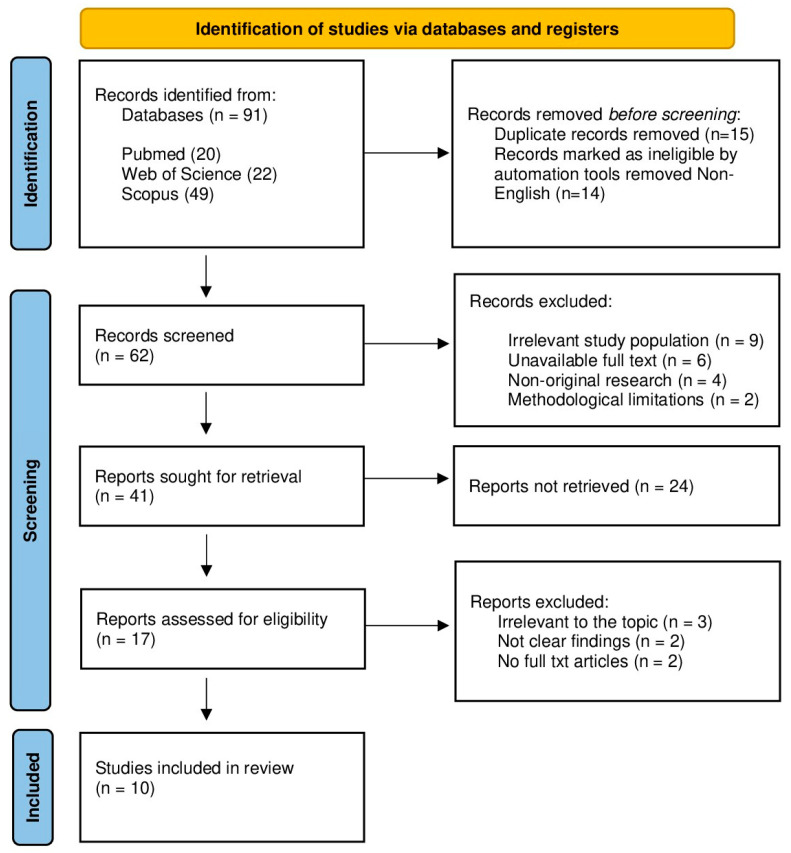
PRISMA 2020 flow diagram summarizing the study selection process, including identification, screening, eligibility assessment, and final inclusion of studies.

The study selection process was carried out in accordance with predefined eligibility criteria and involved four sequential stages: title screening, abstract screening, full-text review, and methodological quality assessment. Eligible populations were defined as adults (≥18 years) with a body mass index (BMI) ≥ 25 kg/m^2^, which, in accordance with WHO classification, includes both overweight (BMI 25.0–29.9 kg/m^2^) and obese (BMI ≥ 30.0 kg/m^2^) individuals. Two independent reviewers were assigned to each stage to ensure objectivity and methodological rigor. Specifically, title and abstract screenings were independently conducted by MEY and TA, whereas DYL and YY were responsible for full-text evaluations and quality assessments. Discrepancies at any step were addressed through structured discussions, and unresolved disagreements were adjudicated by two senior reviewers (VC and GMM), who also made the final inclusion decisions. Literature was identified through three electronic databases, PubMed (20 records), Web of Science (22 records), and Scopus (49 records), resulting in 91 unique studies. After removing duplicates, 62 articles proceeded to full-text screening. Ultimately, 10 studies satisfied the eligibility criteria and were included in the systematic review. Multiple publications from Hernandez-Lepe et al. [11,16,17] were consolidated as a single study for quantitative analysis. Following dataset consolidation and exclusion of insufficient outcome data, 9 studies were included in the meta-analysis. The complete study selection process is illustrated in the PRISMA flow diagram (Figure 1).

### 2.3. Data Extraction

Data extraction was performed independently by researchers (MEY, TA, DYL, and YY) using a standardized form to collect information on author(s), year of publication, country, study design, participant characteristics (age, gender, baseline BMI, health status), intervention details (spirulina dosage and duration, exercise modality and intensity), and primary outcomes (body composition, glycemic and lipid markers, cardiorespiratory fitness, and inflammatory indicators).

Discrepancies were resolved through consultation with reviewers (VC and GMM). When multiple publications originated from the same participant sample, they were treated as a single study and data were extracted once per outcome at the post-intervention time point. For trials with multiple intervention arms sharing a common control group, the control sample size was proportionally divided across comparisons to avoid double counting. Mean differences represent post-intervention between-subgroups differences. Effect sizes were calculated only when sufficient post-intervention data were available; studies lacking necessary information were excluded from quantitative synthesis, and no statistical imputation was performed.

### 2.4. Quality Assessment

**Figure 2:**
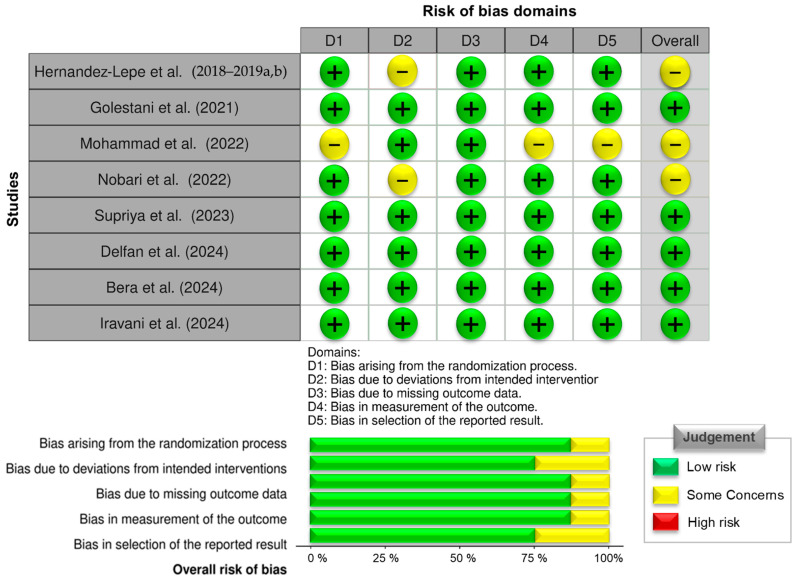
Risk of bias assessment of included studies [11,12,13,16,17,20,21,22,23,24] using the Cochrane Risk of Bias Tool 2.0. Each study was evaluated across five domains: (D1) bias arising from the randomization process, (D2) bias due to deviations from intended interventions, (D3) bias due to missing outcome data, (D4) bias in measurement of the outcome, and (D5) bias in selection of the reported result. Judgements for each domain are color-coded as follows: green (+) indicates low risk of bias, yellow (−) indicates some concerns. The overall risk of bias is also presented for each study.

The methodological quality of included studies was assessed using the Cochrane Risk of Bias Tool 2.0 [18,19], evaluating domains such as randomization, allocation concealment, blinding, handling of missing data, and outcome reporting. Based on these criteria, each study was classified as having low, moderate, or high risk of bias (Figure 2). Ten studies were assessed for risk of bias. One study was excluded from the quantitative synthesis due to insufficient accessible outcome data, resulting in nine studies included in the meta-analysis. The limited number of included studies, variability in sample sizes, and methodological differences may have introduced heterogeneity and limited the generalizability of the findings. These factors were considered during the interpretation of the pooled estimates, and no study was excluded solely based on risk-of-bias classification.

### 2.5. Data Analysis

Meta-analysis was performed using Jamovi (version 2.7.14) with the meta-analysis module for continuous outcomes [25], operating via R (version 4.5; R Core Team, 2025) and the metafor package for meta-analytic computations [26,27]. Mean differences (MDs) were used as the summary effect measure for continuous outcomes and represent post-intervention between-subgroups differences. Random-effects models were fitted to account for between-study variability. Between-study heterogeneity (τ^2^) was estimated using the Hedges estimator. Subgroup analyses were prespecified and considered exploratory. Exercise modalities were categorized a priori based on intervention structure. Cochran’s Q-test and the I^2^ statistic were used to further quantify heterogeneity. Prediction intervals were calculated when τ^2^ exceeded zero to estimate the expected range of true effects in comparable future studies. Potential outliers were assessed using studentized residuals, with values exceeding the Bonferroni-adjusted threshold (two-sided α = 0.05) considered indicative of outlying comparisons. Funnel plot asymmetry was evaluated using rank correlation and regression-based tests, with the standard error of the observed effects used as the predictor. Publication bias was further examined using the trim-and-fill method and the Rosenthal fail-safe N approach. Findings were interpreted cautiously given the small number of trials.

## 3. Results

This systematic review and meta-analysis evaluate the combined effects of spirulina supplementation and structured exercise interventions on body composition, metabolic health, cardiovascular fitness, and inflammatory biomarkers in individuals with obesity or metabolic risk. The following sections present detailed findings from the reviewed studies, highlighting specific outcomes based on distinct study designs and intervention protocols (Table 1). According to Table 1, the combination of spirulina supplementation (1–6 g/day) and structured exercise interventions was associated with changes across multiple physiological domains, including body composition, metabolic health, cardiovascular fitness, and inflammatory regulation.

### 3.1. Anthropometric Outcomes

The RCTs reviewed have indicated that the combination of spirulina supplementation and regular exercise leads to a decrease in BMI and body fat percentage. Larger mean differences were observed in high-intensity protocols such as HIIT, with some studies also reporting preservation or increases in lean mass, in protocols including resistance training.

### 3.2. Glycemic and Lipid Parameters

Interventions combining HIIT and spirulina supplementation were associated with changes in glycemic regulation and lipid metabolism. Reductions in fasting glucose, insulin levels, and HOMA-IR scores were accompanied by changes in insulin-related markers. Concurrently, lipid profiles were associated with favorable shifts, evidenced by decreased LDL and triglyceride concentrations, along with elevated HD levels. These effects were pronounced in protocols of longer duration, when structured exercise was combined with spirulina intake.

### 3.3. Cardiovascular Fitness (VO2peak)

VO2peak values, a key marker of cardiorespiratory fitness, increased across trials, particularly in the HIIT groups. These effects larger mean differences were observed in interventions lasting ≥8 weeks and were with spirulina supplementation.

### 3.4. Inflammatory and Metabolic Biomarkers

Additionally, systemic inflammation were lower, as evidenced by lower CRP, TNF-α, MCP-1, IL-6, and IL-8 levels, while adipokines such as omentin-1, irisin, and spexin exhibited higher concentrations were reported. The magnitude of these effects appeared to vary by dose and intervention duration, with longer interventions generally showing greater effect estimates. These findings suggest that the combination of spirulina supplementation and structured exercise training was associated with improvements in metabolic parameters in metabolically at-risk populations.

### 3.5. Subgroup Analyses

Subgroup findings should be interpreted cautiously due to the limited number of trials. Across subgroup analyses, exercise-containing interventions, particularly combined protocols (R-AEsupp and HIITsupp), tended to show larger effect estimates across BMI, body fat, glucose, VO2max, HDL-C, LDL-C, and TG outcomes, whereas supplementation-only comparisons were associated with smaller and less consistent estimates.

**Figure 3:**
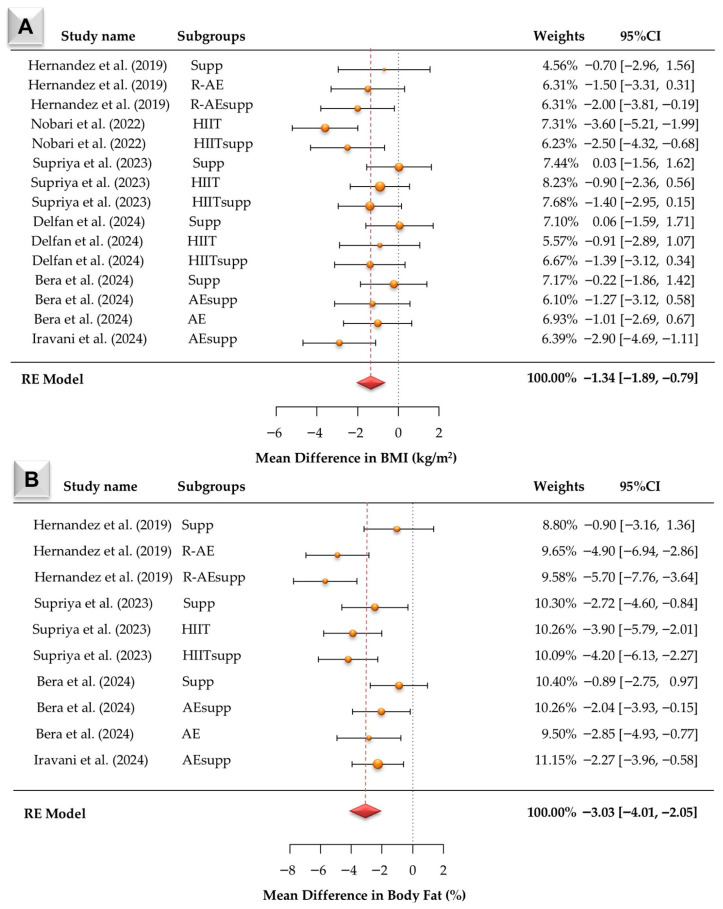
Forest plots of mean differences (MDs) with 95% confidence intervals for body mass index (BMI; Panel (**A**)) [13,16,21,22,23,24] and body fat percentage (Panel (**B**)) [16,22,23,24] in individuals with overweight or obesity from the included studies. Estimates were calculated using a random-effects (RE) model. The red diamond represents the pooled effect size.

According to Figure 3A, fifteen BMI effect sizes were synthesized. Observed mean differences ranged from −3.60 to 0.06 kg/m^2^, with most estimates being negative. The random-effects model produced a pooled mean difference of −1.34 kg/m^2^ (95% CI: −1.89 to −0.79; z = −4.74, *p* < 0.001). Heterogeneity statistics were Q (14) = 21.01 (*p* = 0.101), τ^2^ = 0.41, and I^2^ = 34.95%. This reflects low-to-moderate between-study heterogeneity. The largest negative mean differences were observed in HIIT and HIITsupp, followed by AEsupp, R-AEsupp, and R-AE, whereas supp-only estimates were closer to zero. Funnel plot tests for Figure 6A indicated no publication bias (Begg −0.115, *p* = 0.552; Egger −0.284, *p* = 0.776). Trim-and-fill imputed no studies, and the Rosenthal fail-safe N was 178.

According to Figure 3B, ten body fat percentage effect sizes were synthesized. Observed mean differences ranged from −5.70 to −0.89, and all estimates were negative. The pooled mean difference under the random-effects model was −3.03 (95% CI: −4.01 to −2.05; z = −6.08, *p* < 0.001). Heterogeneity was statistically significant (Q (9) = 22.36, *p* = 0.008), with τ^2^ = 1.49 and I^2^ = 39.2%. This indicates moderate between-study heterogeneity. The largest negative mean differences were observed in R-AEsupp and R-AE, followed by HIITsupp and HIIT, then AE and AEsupp, whereas supp-only comparisons showed the smallest reductions. Funnel plot tests for Figure 6B indicated no publication bias (Begg −0.333, *p* = 0.216; Egger −0.335, *p* = 0.738). Trim-and-fill imputed no studies, and the Rosenthal fail-safe N was 333.

**Figure 4:**
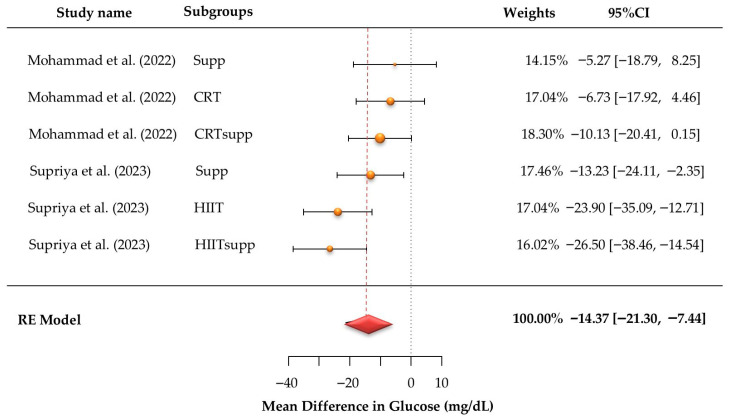
Forest plots of mean differences (MDs) with 95% confidence intervals for glucose levels in individuals with overweight or obesity from the included studies [12,22]. Estimates were calculated using a random-effects (RE) model. The red diamond represents the pooled effect size.

According to Figure 4, six glucose effect sizes were synthesized. Observed mean differences ranged from −26.50 to −5.27, and all estimates were negative. The pooled mean difference under the random-effects model was −14.47 (95% CI: −21.30 to −7.44; z = −4.07, *p* < 0.001). Between-study heterogeneity was not statistically significant (Q (5) = 10.97, *p* = 0.052), with τ^2^ = 40.80 and I^2^ = 54.63%. This reflects moderate heterogeneity across studies. This limits the interpretability of the pooled estimate. The largest negative mean differences were observed in HIITsupp and HIIT, followed by CRTsupp and CRT. Funnel plot tests for Figure 6C indicated no publication bias (Begg −0.067, *p* = 0.960; Egger 0.211, *p* = 0.833). Trim-and-fill imputed no studies, and the Rosenthal fail-safe N was 75 (*p* < 0.001).

**Figure 5:**
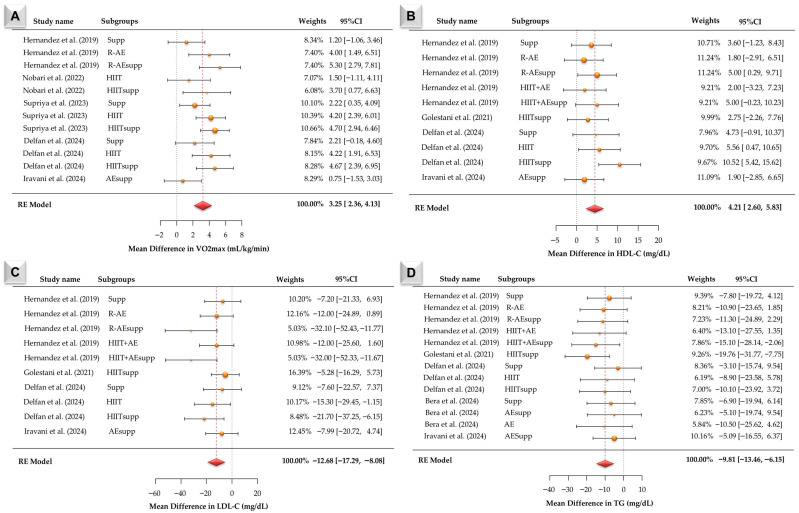
Forest plots of mean differences (MDs) with 95% confidence intervals for VO2max levels (Panel (**A**)) [13,16,21,22,24], HDL-C levels (Panel (**B**)) [13,16,17,20,24] LDL-C levels (Panel (**C**)) [13,16,17,20,24] and TG levels (Panel (**D**)) [13,16,17,20,23,24] in individuals with overweight or obesity from the included studies Estimates were calculated using a random-effects (RE) model. The red diamond represents the pooled effect size.

**Figure 6:**
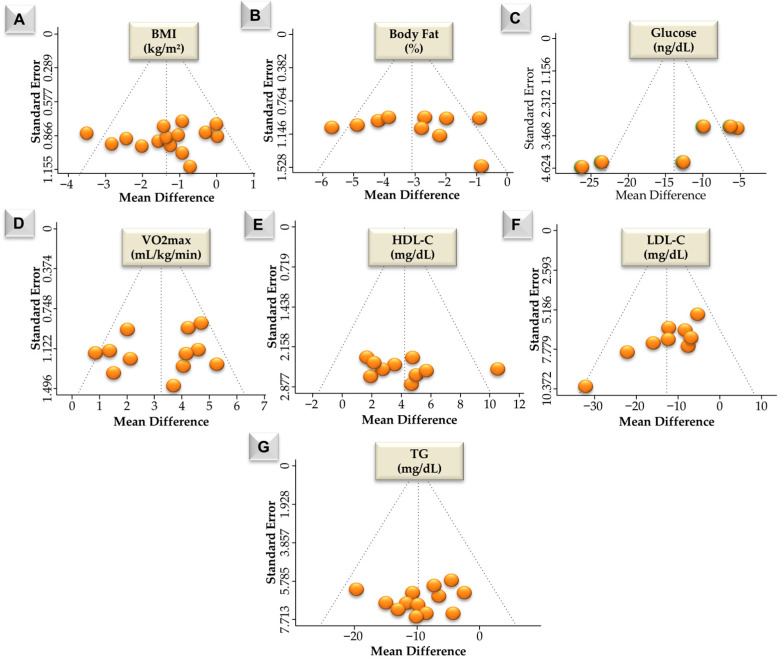
Funnel plots of mean differences (MDs) for BMI levels (**A**), body fat percentage (**B**), glucose (**C**) levels, VO2max levels (**D**), HDL-C levels (**E**), LDL-C levels (**F**), and TG levels (**G**). Each orange circle represents an individual effect size. The x-axis displays the effect estimate (MD), and the y-axis shows the standard error. The vertical dotted line indicates the pooled effect under the random-effects model, and the diagonal lines denote the pseudo 95% confidence limits. Abbreviations: BMI, body mass index; HDL-C, high-density lipoprotein cholesterol; LDL-C, low-density lipoprotein cholesterol; TG, triglycerides. The visual representations of publication bias for BMI, body fat percentage, glucose, VO2max, HDL-C, LDL-C, and TG are provided as funnel plots in Figure 6 (Panels (**A**–**G**), respectively).

According to Figure 5A, twelve VO2max effect sizes were synthesized. Observed mean differences ranged from 1.20 to 5.30, and all estimates were positive. The pooled mean difference under the random-effects model was 3.25 (95% CI: 2.36 to 4.13; z = 7.21, *p* < 0.001). Heterogeneity was statistically significant (Q (11) = 20.23, *p* = 0.042), with τ^2^ = 1.10 and I^2^ = 35.95%. This indicates moderate between-study heterogeneity. The 95% prediction interval ranged from 0.95 to 5.55. The largest positive mean differences were observed in R-AEsupp and HIITsupp, followed by R-AE and HIIT, whereas supp and AEsupp comparisons showed smaller. Funnel plot tests for Figure 6D indicated no publication bias (Begg −0.137, *p* = 0.536; Egger −0.391, *p* = 0.696). Trim-and-fill imputed two studies, and the Rosenthal fail-safe N was 417. According to Figure 5B, ten HDL-C effect sizes were synthesized. Observed mean differences ranged from −1.80 to 10.52, with most estimates being positive. The pooled mean difference under the random-effects model was 4.21 (95% CI: 2.60 to 5.83; z = 5.11, *p* < 0.001). Heterogeneity was not statistically significant (Q (9) = 9.37, *p* = 0.404), with τ^2^ = 0.25 and I^2^ = 3.75%. This indicates minimal between-study heterogeneity. The largest HDL-C increases were observed in HIITsupp, followed by R-AEsupp and HIIT + AEsupp, whereas HIIT and supp showed moderate effects and R-AE, HIIT + AE, and AEsupp showed smaller estimates. Funnel plot tests for Figure 6D indicated no publication bias (Begg −0.137, *p* = 0.536; Egger −0.391, *p* = 0.696). Trim-and-fill imputed two studies, and the Rosenthal fail-safe N was 417.

According to Figure 5C, ten LDL-C effect sizes were synthesized. Observed mean differences ranged from −34.10 to −7.20, and all estimates were negative. The pooled mean difference under the random-effects model was −12.68 (95% CI: −17.29 to −8.08; z = −5.40, *p* < 0.001). Heterogeneity was not statistically significant (Q (9) = 11.69, *p* = 0.231), with τ^2^ = 2.08 and I^2^ = 3.75%. This indicates low between-study heterogeneity. The 95% prediction interval ranged from −18.51 to −6.89. The LDL-C reductions were greatest in R-AEsupp and HIIT + AEsupp, followed by HIIT, HIITsupp, R-AE, and HIIT + AE, whereas supp and AEsupp comparisons showed smaller or less consistent changes. Funnel plot tests for Figure 6F suggested asymmetry (Begg −0.719, *p* = 0.004; Egger −3.051, *p* = 0.002). Trim-and-fill imputed one study, and the Rosenthal fail-safe N was 120. According to Figure 5D, thirteen effect sizes were synthesized. Observed mean differences ranged from −10.16 to −6.19, and all estimates were negative. The pooled mean difference under the random-effects model was −9.81 (95% CI: −13.46 to −6.15; z = −5.26, *p* < 0.001). Heterogeneity was not statistically significant (Q (12) = 6.00, *p* = 0.916), with τ^2^ = 0 and I^2^ = 0%. This indicates no observed between-study heterogeneity. The 95% prediction interval ranged from −13.46 to −6.15. The largest TG reductions were observed in HIITsupp and HIIT + AEsupp conditions, followed by R-AE– and AE-based conditions, whereas supp-only comparisons were generally closer to zero. Funnel plot tests for Figure 6F indicated no publication bias (Begg −0.077, *p* = 0.765; Egger −0.052, *p* = 0.959). Trim-and-fill imputed no studies, and the Rosenthal fail-safe N was 120.

## 4. Discussion

The present meta-analysis suggests that R-AE, R-AEsupp, HIIT, and HIITsupp improvements across several outcomes in body composition and metabolic outcomes, whereas supp-only comparisons exhibited smaller and less consistent estimates. Body fat percentage reductions were observed across all subgroups, with larger mean differences in R-AEsupp and R-AE subgroups. Glucose reductions were greatest in HIITsupp and HIIT, with moderate between-study heterogeneity (I^2^ = 54.63%). VO2max increases were largest in R-AEsupp and HIITsupp, followed by R-AE and HIIT, while supp-only effects were smaller. The HDL-C level increases and LDL-C and TG level reductions were also observed, with larger effect estimates generally occurring in R-AEsupp and HIITsupp compared with supp-only conditions. However, these subgroup findings should be considered exploratory

Structured exercise protocols were associated with reductions in BMI and body fat percentage, with the largest mean differences observed in HIIT and HIITsupp for BMI and in R-AEsupp and R-AE for body fat percentage, whereas supp-only comparisons showed smaller estimates. These findings are partially consistent with Hossein et al. [28], who reported that circuit resistance training combined with spirulina influenced appetite regulation and energy balance, and with Sadeghipour et al. [29], who observed improvements in muscle-related adaptations alongside reductions in adiposity following spirulina supplementation. Bera et al. [23] also reported decreases in body fat after spirulina intake in overweight young men, although the magnitude of change varied across studies. Bohórquez-Medina et al. [30] noted that spirulina may exert favorable effects on metabolic disturbances associated with obesity and dyslipidemia, while emphasizing inter-individual variability. Weston et al. [10] highlighted the impact of high-intensity interval training on body composition; however, HIIT and HIITsupp were associated with larger reductions in anthropometric outcomes, followed by R-AEsupp and R-AE. Potential contributions of spirulina, including its protein content and antioxidant properties [31], may be associated with metabolic regulation; however, the observed effects appeared to depend on the specific intervention protocol rather than supplementation alone [32].

Reductions in glucose were most pronounced in HIITsupp and HIIT, followed by CRTsupp and CRT, whereas supp-only comparisons showed smaller estimates. Although the pooled mean difference was significant, moderate between-study heterogeneity (I^2^ = 54.63%) indicates variability in effect magnitude. Funnel plot tests showed no evidence of small-study effects (Begg −0.067, *p* = 0.960; Egger 0.211, *p* = 0.833). Evidence suggests that HIIT may improve glycemic regulation through increased skeletal muscle glucose uptake and insulin sensitivity, potentially involving enhanced GLUT4-mediated glucose transport (translocation and/or expression) and improvements in mitochondrial biogenesis/content and oxidative capacity [33,34]. Spirulina has been associated with insulin-modulating and hypoglycemic properties, potentially mediated by phycocyanin content, antioxidant activity, and attenuation of inflammatory pathways linked to insulin resistance [24]. High-intensity protocols may also induce AMPK activation and glycogen depletion–repletion dynamics, which can contribute to improved glucose disposal [35,36]. Furthermore, insulin sensitivity and endocrine adaptations may be considered within broader lifestyle-based evidence. Changes in insulin dynamics and markers such as vitamin D status have been reported following combined dietary and exercise interventions in individuals with obesity [37]. These findings provide contextual background for interpreting the observed associations.

Improvements in VO2max were observed across subgroups, with the largest mean differences in R-AEsupp and HIITsupp, followed by R-AE and HIIT, whereas supp-only comparisons showed smaller increases and, in some cases, confidence intervals overlapping zero. Heterogeneity was moderate (I^2^ = 45.95%), and the prediction interval remained positive, indicating a consistent direction of effect across studies. These findings are concordant with established evidence that HIIT enhances cardiorespiratory capacity through central and peripheral adaptations, including increased stroke volume, improved endothelial function, enhanced mitochondrial biogenesis, and greater oxidative enzyme activity [33,34]. Resistance–aerobic protocols similarly contribute to aerobic improvements via augmented muscle capillarization and improved oxygen extraction [38,39]. Spirulina supplementation has been proposed to support aerobic performance through antioxidant effects, modulation of nitric oxide availability, and improved hemoglobin function, particularly under higher-intensity training conditions [31].

Bera et al. [23] also reported that spirulina supplementation improved HDL-C, decreased TG, and enhanced overall body composition. Torres-Duran et al. [40] emphasized that spirulina may reduce LDL while increasing HDL, potentially lowering cardiovascular risk. Rather than functioning as an independent lipid regulator, spirulina appears to exert its greatest effects when paired with metabolically demanding interventions like HIIT. The positive effects of HIIT on lipid profile are known to occur through enhanced fat oxidation, improved insulin sensitivity, and elevated lipoprotein lipase activity [41]. Spirulina, on the other hand, contains polyphenols, phycocyanin, and γ-linolenic acid, which help reduce oxidative stress and inflammation, thereby supporting lipid metabolism [42]. Collectively, these mechanisms highlight the potential of spirulina as a complementary strategy alongside pharmacological therapies in individuals at risk for dyslipidemia. From a clinical perspective, the findings are consistent with exercise-based dyslipidemia management guidelines advocated by the American College of Sports Medicine (ACSM) and the European Society of Cardiology [40]. However, it remains essential to personalize exercise type and intensity based on individual characteristics. Golestani et al. [20] observed lipid profile improvements following HIIT combined with spirulina, noting that the magnitude of change varied according to age, metabolic status, and dietary habits. Therefore, multi-component interventions based on HIIT and spirulina supplementation may represent a complementary strategy pending confirmation in larger trials. In the present meta-analysis, HDL-C increased (MD = 4.21, 95% CI: 2.60 to 5.83), with the largest subgroup estimate observed in HIITsupp, whereas supp-only and R-AE subgroups showed smaller effects. LDL-C was reduced (MD = −12.68, 95% CI: −17.29 to −8.08), with the greatest reductions in R-AEsupp and HIIT + AEsupp. TG levels were also reduced (MD = −9.81, 95% CI: −13.46 to −6.15), with the largest subgroup estimates in HIITsupp and HIIT + AEsupp, while supp-only comparisons were closer to zero. These subgroup distributions parallel prior reports indicating that spirulina-related lipid modifications are more evident when supplementation is combined with higher-intensity training stimuli, while also reflecting the established lipid-modulatory effects of HIIT [20,22,43,44]. The included studies involved adults with overweight or obesity, with modest sample sizes and limited demographic diversity. Therefore, generalizability to broader clinical populations remains uncertain.

### Limitations

Several limitations should be considered. The number of eligible randomized trials per outcome was limited, with modest sample sizes and relatively short intervention durations (typically 6–12 weeks), restricting long-term inference and external validity. Variability in spirulina dosage, exercise modality and intensity (HIIT, R-AE, CRT), and intervention structure (single- vs multi-component protocols), and baseline metabolic characteristics, and control conditions likely contributed to between-study heterogeneity, particularly for glycemic outcomes. Reporting of dietary intake and energy balance was incomplete in several trials, and body composition assessment methods were not consistently described. These methodological differences may have affected effect estimate precision, comparability, and the consistency of pooled results.

## 5. Conclusions

This systematic review and meta-analysis indicate that spirulina supplementation combined with structured exercise was associated with changes in body composition, glycemic measures, lipid parameters, and VO2max in individuals with overweight or obesity. Pooled analyses were associated with reductions in BMI, body fat percentage, fasting glucose, LDL-C, and triglycerides, and increases in HDL-C and VO2max. The R-AEsupp and HIITsupp showed larger estimates than supp-only comparisons; however, variability in intervention characteristics and study populations limits inference regarding the magnitude and consistency of these associations. In addition, exercise and combined exercise–spirulina conditions were associated with larger effect estimates than spirulina alone.

## Figures and Tables

**Table 1:** The effects of spirulina supplementation and exercise training on body composition, metabolic health, cardiovascular fitness, and ınflammation: a summary of randomized controlled trials.

Authors	Study Desing	Anthropometric Measurements	Glycemic and Lipid Metabolism Markers	Cardio Pulmonary Fitness	Metabolic Biomarkers and Inflammatory Molecules
Hernandez-Lepe et al. (2018–2019a,b) * [11,16,17]	Experimental Desing: Double-blind, randomized, and crossover controlled clinical trial Participant: Sedentary men with overweight or obesity (N = 52; BMI ≥ 25) Spirulina Dose: 4.5 g/day/kg for 6 weeks Exercise Training: 6 weeks, 5 days/week combined resistance aerobic exercise or HIIT (50–90% HRR)	BMI ↓ Body Fat % ↓ Lean Mass (kg) (NM)	LDL-C ↓ TG ↓ HDL-C ↑	VO2max (mL/kg/min) ↑	(NM)
Golestani et al. (2021) [20]	Experimental Design: Randomized, quasi-experimental controlled, single-blind trial Participant: Overweight and obese women (N = 20; BMI ≥ 25) Spirulina Dose: 1 g/day for 4 weeks Exercise Training: 4-week HIIT (RAST protocol, 100% max speed), 3 days/week	Body Fat % ↓ BMI (NM) WHR (NM)	TC (NM) TG (NM) LDL-C (NM) HDL-C (NM)	(NM)	Nesfatin-1 ↑ Omentin-1 ↑
Mohammad et al. (2022) [12]	Experimental Desing: Randomized and controlled clinical trial Participant: Sedentary men with overweight or obesity (N = 60; BMI ≥ 25) Spirulina Dose: 1 g/day for 8 weeks Exercise Training: 8-week CRT	(NM)	Glucose (mg/dL) ↓	(NM)	Adipolin: ↑ Apelin: ↓
Nobari et al. (2022) [21]	Experimental Desing: randomized and controlled clinical trial Participant: Sedentary men with overweight or obesity (N = 30; BMI ≥ 25) Spirulina Dose: 6 g/day for 8 weeks Exercise Training: 8-week HIIT (90% HRR).	BMI ↓ Body Fat % ↓ Lean Mass (kg) (NM)	(NM)	VO2max (mL/kg/min) ↑	(NM)
Supriya et al. (2023) [22]	Experimental Desing: Randomized and controlled clinical trial Participant: Sedentary men with overweight or obesity (N = 44; BMI ≥ 25) Spirulina Dose: 6 g/day for 12 weeks Exercise Training: 12-week HIIT: Week 1–12 (65–95% VO2max, progressive overload)	BMI ↓	LDL-C ↓ TG ↓ HDL-C ↑	VO2max (mL/kg/min) ↑	CRP: ↓ TNF- α: ↓ Sema3C: ↓ MCP-1: ↓ IL-6: ↓ IL-8: ↓
Delfan et al. (2024) [13]	Experimental Desing: Randomized and controlled clinical trial Participant: Sedentary men with obesity (N = 44; BMI ≥ 30) Spirulina Dose: 6 g/day for 12 weeks Exercise Training: 12-week HIIT: 65–95% VO2max, progressive load	BMI ↓ Body Fat % ↓ Lean Mass (kg) ↑	Insulin (μU/mL) ↓ HOMA-IR ↓ Glucose (mg/dL) ↓	VO2max (mL/kg/min) ↑	Asprosin ↓ Lipocalin-2 ↓ Omentin-1 ↑ Irisin ↑ Spexin ↑
Bera et al. (2024) [23]	Experimental Design: Randomized controlled trial Participant: Sedentary male overweight college students (N = 32; BMI 25–29.9) Spirulina Dose: 5 g/day for 12 weeks Exercise Training: 12-week moderate-intensity aerobic training, 4 days/week (55–75% HRmax)	BMI ↓ Body Fat % ↓	TG ↓	(NM)	(NM)
Iravani et al. (2024) [24]	Experimental Design: Randomized controlled trial Participant: Overweight and obese women (N = 20; BMI ≥ 25) Spirulina Dose: 8 g/day for 8 weeks Exercise Training: 8-week moderate-intensity aerobic exercises	Weight BMI Body Fat % ↓ Muscle Strength ↑	LDL-C ↓ TG ↓ HDL-C ↑	VO2max (mL/kg/min)	(NM)

BMI: Body Mass Index; N: the number of participants included in each study; HIIT: High-Intensity Interval Training; HRR: Heart Rate Reserve; CRP: C-Reactive Protein; CRT: Cyclical Resistance Training; HDL: High-Density Lipoprotein;; HOMA-IR: Homeostatic Model Assessment for Insulin Resistance; IL-6: Interleukin-6; IL-8: Interleukin-8; LDL: Low-Density Lipoprotein; MCP-1: Monocyte Chemoattractant Protein-1; ↓: Decrease; ↑: increase; NM: Not Mentioned; TNF-α: Tumor Necrosis Factor-alpha; VO2max: Peak Oxygen Uptake. *: Study by Hernandez-Lepe et al. [11,16,17] were considered as multiple reports from the same participant sample and were treated as a single study in the meta-analysis.

## Data Availability

The data presented in this study are available upon request from the corresponding authors.

## Supplementary Materials

The following supporting information can be downloaded at https://www.mdpi.com/article/10.3390/jcm15062137/s1: Supplementary Material S1: PRISMA 2020 Checklist [15].

## Abbreviations

The following abbreviations are used in this manuscript:

BMI	Body mass index
CRP	C-reactive protein
CRT	Cyclical resistance training
HDL-C	High density lipoprotein cholesterol
LDL-C	Low density lipoprotein cholesterol
TG	Triglyceride
HIIT	High-intensity interval training
HRR	Heart rate reserve
HOMA-IR	Homeostatic model assessment for insulin resistance
IL-6	Interleukin-6
IL-8	Interleukin-8
MCP-1	Monocyte chemoattractant protein-1
NM	Not mentioned
TNF-α	Tumor necrosis factor-alpha
VO2max	Peak oxygen uptake
